# Mapping the brain's orchestration during speech comprehension: task-specific facilitation of regional synchrony in neural networks

**DOI:** 10.1186/1471-2202-5-40

**Published:** 2004-10-24

**Authors:** Markus Härle, Brigitte S Rockstroh, Andreas Keil, Christian Wienbruch, Thomas R Elbert

**Affiliations:** 1 Department of Psychology, University of Konstanz, Germany

## Abstract

**Background:**

How does the brain convert sounds and phonemes into comprehensible speech? In the present magnetoencephalographic study we examined the hypothesis that the coherence of electromagnetic oscillatory activity within and across brain areas indicates neurophysiological processes linked to speech comprehension.

**Results:**

Amplitude-modulated (sinusoidal 41.5 Hz) auditory verbal and nonverbal stimuli served to drive steady-state oscillations in neural networks involved in speech comprehension. Stimuli were presented to 12 subjects in the following conditions (a) an incomprehensible string of words, (b) the same string of words after being introduced as a comprehensible sentence by proper articulation, and (c) nonverbal stimulations that included a 600-Hz tone, a scale, and a melody. Coherence, defined as correlated activation of magnetic steady state fields across brain areas and measured as simultaneous activation of current dipoles in source space (Minimum-Norm-Estimates), increased within left- temporal-posterior areas when the sound string was perceived as a comprehensible sentence. Intra-hemispheric coherence was larger within the left than the right hemisphere for the sentence (condition (b) relative to all other conditions), and tended to be larger within the right than the left hemisphere for nonverbal stimuli (condition (c), tone and melody relative to the other conditions), leading to a more pronounced hemispheric asymmetry for nonverbal than verbal material.

**Conclusions:**

We conclude that coherent neuronal network activity may index encoding of verbal information on the sentence level and can be used as a tool to investigate auditory speech comprehension.

## Background

One key function of the cerebral cortex involves the integration of elements into a percept that separates them from the background. In this process, changes in cortical networks are formed and modified by experience through the simultaneous excitation of groups of neurons [1-3]. These "long-range connections formed by excitatory cortical neurons" [[4] p.3] are considered the anatomical substrate of this integrative capability. This integration has been modeled in detail for the visual system [e.g., [4]] and similar principles should also describe other sensory functions such as auditory speech perception and comprehension. This assumption was tested in the present study by probing patterns of co-activation within and across hemispheres during the processing of verbal and nonverbal acoustic material. Intra-hemispheric co-activation was taken as a large-scale measure of functional network activation, and coherence of oscillatory electromagnetic activity served as measure of co-activation in time. Coherence is defined as the correlated activity between two locations within a distinct frequency range.

Event-related brain responses, traditionally used in the study of cognitive processes, have been found to result from regional perturbations in ongoing brain activities in a self-organizing system rather than constituting a response set from an otherwise silent system. For example, Makeig and coworkers [5-7] showed that event-related potentials (ERP) must be viewed as perturbations in the oscillatory dynamics of the ongoing EEG. The response of successively activated groups of neurons is governed by an attractor, which means that different neuron groups, one after the other, contribute to large-scale changes in the magnetic field that move across brain areas, indicating spatio-temporal changes on a macroscopic level. The basin of attraction guarantees robustness of the propagating synchrony. Therefore, the activation of functional cortical networks may best be determined by examining the pattern of dynamic co-activation of groups of neurons [8,9]. As such, whenever neuronal cell assemblies fire 'in phase' the amplitude of oscillatory activity will increase.

On a macroscopic level, oscillatory coupling between large neuronal populations can be examined by externally driving the nervous system using oscillatory stimulation and then measuring the regional coherence of the electromagnetic activity [10]. Amplitude modulation of the stimuli induces the oscillatory pattern of the Steady-State-Response (SSR). For auditory stimuli the SSR is most prominent at modulation frequencies around 40 Hz [11]. Patel & Balaban [12] assessed the synchronization of the magnetoencephalographic SSR at this frequency over time (i.e., coherence) in order to investigate neural correlates of musical comprehension. When the stimulus sequences formed a percept (a melody relative to random sequence), coherence increased between left posterior and right frontal nodes. Similarly, Srinivasan et al [13] found increased inter- and intra-hemispheric coherence in the visual SSR when subjects consciously recognized visual stimuli in their field of view. Coherence measures have also been employed in the investigation of complex networks involved in the processing of nouns [14,15], music [16], the perception of Necker cube reversals [17], and in the acquisition of contingencies in a conditioning paradigm [18].

The present study investigated coherence patterns of the auditory evoked magnetic Steady-State-Field (SSF), specifically coherence among SSF-generators within and across hemispheres, as a measure of neural networks involved in speech comprehension. If, as we hypothesized, the *comprehension *of speech was related to the activation of neuronal assemblies in the left hemisphere, then we should see increased coherence in this region with the recognition of a meaningful sentence as compared to an incomprehensible string of sounds. We further hypothesized that meaningful verbal stimuli should be processed differently from musical melodies. That is to say, verbal material should affect the coherence of electromagnetic signals more in the left than in the right hemisphere whereas listening to a nonverbal complement of a meaningful sentence like a melody will activate more right- than left-hemispheric neuronal networks and influence coherence patterns involving the right hemisphere. Given that language and music share components, we assumed only a relative dominance in the interconnection of networks toward left- or right-hemispheric activity.

## Results and discussion

The present study studied co-activated cortical networks involved in speech comprehension by using auditory steady-state (41.5-Hz amplitude modulated) stimuli and measuring the coherence of generator activity of the magnetic steady state response. Steady-state stimulus modulation were used for a sentence, which – following a German play-of-words – was first presented as an incomprehensible string of sounds, but became a comprehensible sentence after the sentence's meaning was explained to the subjects and was properly articulated. In addition to verbal stimuli, non-verbal stimuli were also studied which included a 600-Hz tone, a scale, and a melody-like combination of the scales' tones. The present analysis of SSF coherence in the source space (see methods) extended previous approaches [12], which employed SSR in the signal space to disclose networks involved in auditory perception.

**Figure 1:**
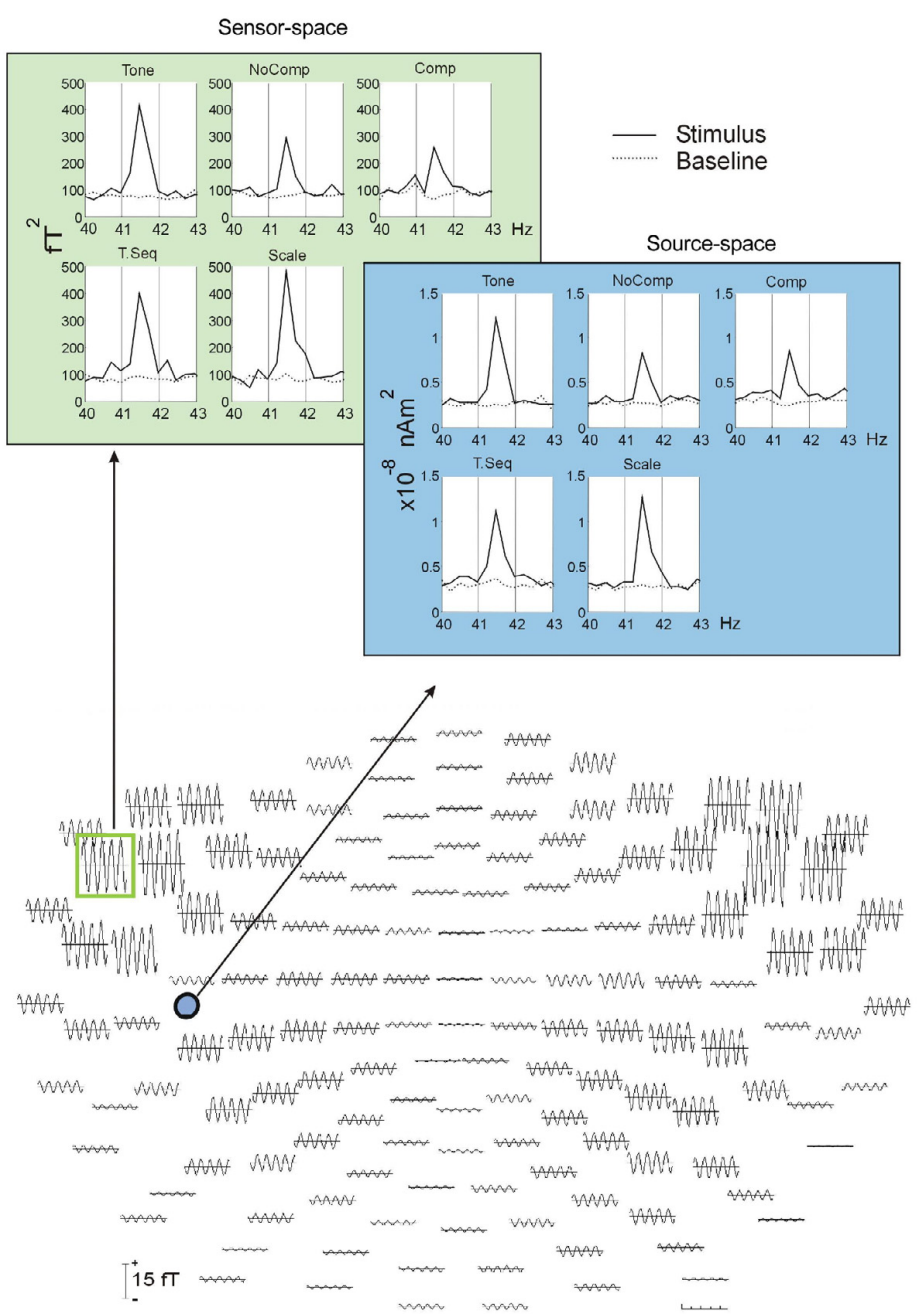
Spectral power and topography of the Steady-State-Field (averages of 12 subjects). Bottom graph: topography of the magnetic SSF evoked by the 600 Hz tone (top view, nose up). Upper left graph (green box): spectral power of the magnetic SSF at a selected sensor (left anterior, indicated by green frame) for all conditions and the baseline. Upper right graph: (blue box): spectral power of the magnetic SSF in the source space (MNE) for one selected dipole location. This dipole is approximately located in the area of the left auditory cortex and is indicated by a blue filled circle. Conditions: Tone = 600 Hz tone, NoComp = incomprehensible word string, Comp = word string after comprehension as sentence, T.seq = melody-like sequence of tones, Scale = tone scale).

**Figure 2:**
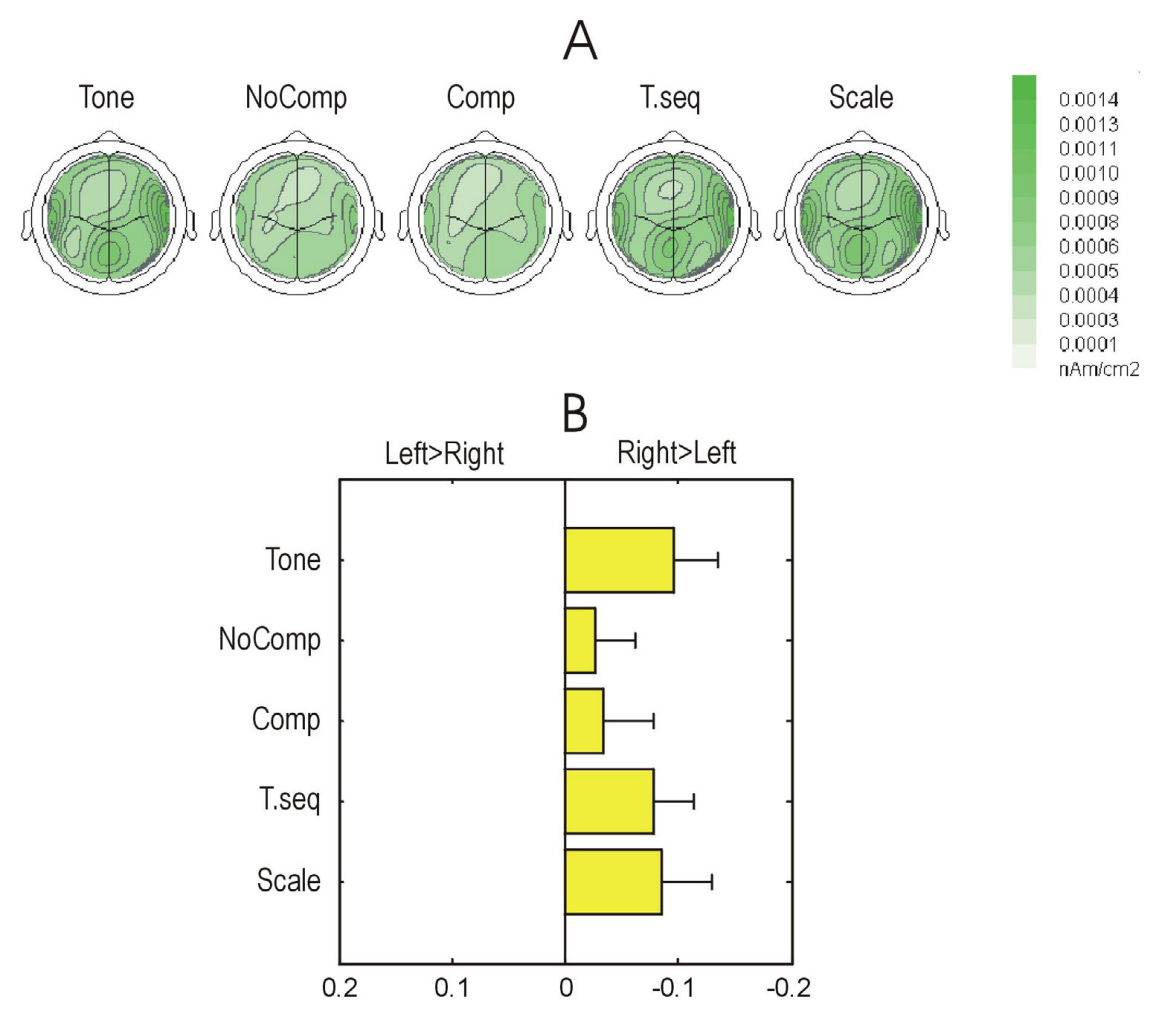
A: Topographical distributions of the SSF in source space (averages of 12 subjects) separately for the five conditions; Orthographical top view, spherical spline interpolation, nose indicated by a small triangle. Each contour-line represents 0.000125 nAm/cm^2 ^(rounded values at scale). Dark grey indicates higher values of activity. B: Asymmetry of activity in the source space as indicated by the Laterality Index (all left- minus all right-hemispheric MNE amplitude values divided by their sum), averaged across subjects. Positive values indicate left lateralized activity and negative values indicate right-lateralized activity. T-bars represent the standard error of the mean). Tone = 600 Hz tone, NoComp = incomprehensible word string, Comp = word string after comprehension as sentence, T.seq= melody-like sequence of tones, Scale = tone scale.

Figure 1 (lower part) gives an example of the evoked magnetic 41.5-Hz SSF, averaged for the tone condition at the 148 sensors across the 12 subject. The sinusoidal 41.5 Hz oscillation is evident at all 148 sensors and a change in polarity over temporal areas suggests generator sources in the temporal cortices of each hemisphere. The Fourier Transform confirms the peak at the modulation frequency of 41.5 Hz for all stimulus-conditions in the sensor space (Fig. 1, upper left graph) and in the source space (mid-right graph in Fig. 1; illustrated for a selected dipole in the expected generator structure of the SSF, as indicated by the filled circle). No such peak was observed during the baseline. A comparison of the grand averages of the power spectra in sensor and source space (see Fig. 2A) demonstrates that conversion using the Minimum Norm Estimate (see methods) preserves the basic profile across conditions.

As expected for acoustic stimulation, overall *MNE amplitudes *were most pronounced in auditory areas of both hemispheres, with a varying degree of laterality. For the Laterality Index (see methods and Fig. 2B) an interaction of CONDITION × HEMISPHERE (F(4,44) = 3.06, p < 0.05, ε = 0.69) verified that nonverbal conditions as compared to the verbal ones induced a more pronounced asymmetry with more activity in the right compared to the left hemispheres (for the main effect of HEMISPHERE, F(1, 11) = 3.33, p < 0.1, and for the main effect of CONDITION, F(4,44) = 12.65, p < 0.0001, ε = 0.57). Planned comparisons confirmed significant effects of HEMISPHERE only for the nonverbal conditions (tone, t(1,11) = 4.5, p < 0.0001, scale, t(1,11) = 4.3, p < 0.000, and melody-like tone sequence, t(1,11) = 3.8, p < 0.0005).

**Figure 3:**
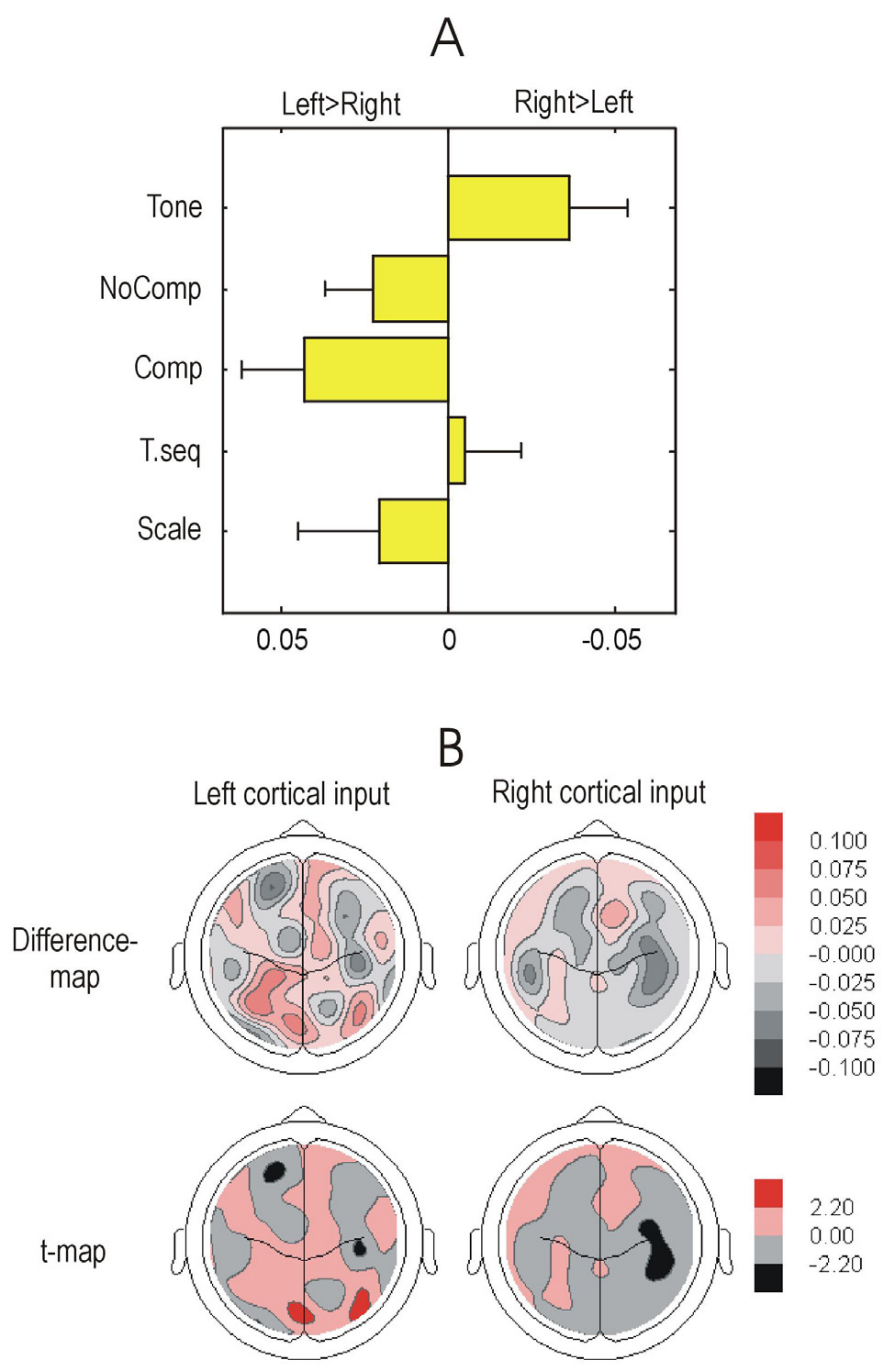
Topography of coherence measure in source space (averages of 12 subjects). A: Asymmetry of intra-hemispheric coherence measures as indicated by the Laterality Index (all left- minus all right-intra-hemispheric coherence values divided by their sum) for the five conditions. Positive values indicate left-lateralized coherence and negative values indicate right-lateralized coherence. T-bars represent the standard error of the mean). Tone = 600 Hz tone, NoComp = incomprehensible word string, Comp = word string after comprehension as sentence, T.seq= melody-like sequence of tones, Scale = tone scale. B: Difference-maps and t-maps of the coherence values comparing the two verbal conditions (Comp minus NoComp). Maps are shown in 110° top view and are spherical spline interpolated; nose indicated by triangle. The left graphs show the coherence between the area of the left auditory cortex and all 77 dipole-locations (left cortical input), the right graphs display the coherence between the area of the right auditory cortex and all 77 dipole-locations (right cortical input). For difference-maps, each contour-line represents a step of 0.025, without units. Pink and red colours illustrate higher coherence values after sentence comprehension (Comp>NoComp), grey and black colours illustrate higher coherence values for the incomprehensible word string (Comp<NoComp). For t-maps, significant differences are shown at the 5% level in red (Comp>NoComp) and black (Comp<NoComp).

Intra-hemispheric *coherence *was specifically affected by conditions (CONDITION × HEMISPHERE, F(4, 44) = 3.72, p < 0.05, ε = 0.46): As illustrated in Fig. 3A for the Laterality Index, higher intra-hemispheric coherence in the left than in the right hemisphere was induced when the string of words became a comprehensible sentence (planned comparison: t(1, 11) = 2.7, p < 0.01), whereas the tone induced higher intra-hemispheric coherences in the right as compared to the left hemisphere (t(1, 11) = 2.3, p < 0.05). The main effect of CONDITION was significant for intra-hemispheric coherence (F(4,44) = 8.35, p < 0.001, ε = 0.62) and inter-hemispheric coherence (F(4,44) = 10.79, p < .001, ε = 0.61) indicating higher coherence was induced by nonverbal than by verbal conditions. Since inter-hemispheric coherence may depend on the different generator strength, which was higher in the right than in the left hemisphere, the coherence measures were normalized in order to compensate for an effect of the signal to noise ratio. For normalization, the inter-hemispheric coherence measures were divided by the intra-hemispheric coherence measure of each condition. Still, a main effect CONDITION (F(4,44) = 12.1, p < 0.0001, epsilon = 0.76) indicates that coherence was larger for nonverbal than for verbal conditions.

Given that the major goal was to depict network signatures specifically involved in sentence comprehension, we applied an ANOVA to compare the coherence measure of the two verbal conditions. These were identical with respect to the physical stimulation, but differed in meaningful comprehension. For intra-hemispheric coherence a significant interaction involving CONDITION × HEMISPHERE × GRADIENT (F(1, 11)= 7.37, p < 0.05) reflected a relatively higher coherence in the left-posterior area after the string of words had been made comprehensible by explaining the sentence's meaning as opposed to the higher coherence in the right-posterior area for the incomprehensible word string. Profiles of intra- and inter-hemispheric coherence were similar, thereby resulting in similar statistical power for the CONDITION effect. This cannot be explained simply by a reduced signal-to-noise ratio in the verbal conditions, because normalized values show the same effect. We rather assume that increased laterality varies with decreased inter-hemispheric communication (coherence).

Inter-hemispheric coherence between dipoles located in the left (left cortical input) and right (right cortical input) auditory cortex and the remaining dipole sites are characterized (Fig. 3B) by larger coherence of activity across areas including the left auditory, occipital and right-posterior regions in response to the comprehensible sentence relative to the incomprehensible word string.

Considering coherent activity, i.e., synchronized oscillations between spatially distributed maps, as the representation of a percept, we followed Makeig et al. [6,7] who discuss evoked activity in terms of oscillatory perturbations, i.e., alteration of synchrony in ongoing activity. The comparison of two conditions with identical physical stimulation but different degrees of integration into a percept revealed that the synchronicity of auditory SSF increased among areas in the posterior left-temporal and right-occipital cortex when a sentence was comprehensible compared to the same material being incomprehensible. This suggests that a network was activated when an intelligible sentence was being processed. This assumption is in line with previous research in which a left-posterior activity focus was found during semantic processing [19-23], a left lateralized auditory-conceptual interface was localized at the temporal-parietal-occipital junction [24], and an occipital focus of oscillatory activity found for the processing of (visually presented) content words relative to verbs [25].

Whereas Scott et al. [26] reported an increase in regional cerebral blood-flow in the anterior part of the left superior temporal sulcus for intelligible sentences compared to acoustically equivalent non-intelligible sentences, the present results indicated such a pattern – enhanced left-anterior coherence – to be induced by the incomprehensible string of words (see Fig. 3B). At this point, hypotheses to resolve this discrepancy must remain provisional. However, it seems possible, that the speech-like – though incomprehensible – stimuli activated syntactical processing which has been associated with frontal activity [27]. In addition, the attempt to determine a syntactical structure has been found to activate the right temporal area [39] which would be in line with the right temporal coherence found for the present condition of incomprehensible word string processing (see Fig. 3B). Patel and Balaban [12] discussed increased coherence between the left posterior and right frontal areas for melody-like stimuli as a correlate of integrative processing of local and global pitch information. Thus, it seems possible that in our study the condition of incomprehensible word string similarly activated pitch processing.

Finally, there is the possibility that the order of stimulus presentations may have affected the results. While counterbalancing was not possible for the specific verbal stimulus condition (see methods), we would not have expected order effects to be large since similar temporal dynamics were not observed for the nonverbal conditions. However, an effect of time cannot be ruled out as steady state responses and their generator activity were largest for a simple 600-Hz tone which was presented first.

SSF were larger for the nonverbal conditions (tone, scale, melody) than for the verbal material, particularly in the right hemisphere. While right-hemispheric processing of tonal perception has frequently been reported [28-31], the general dominance of right-hemispheric SSF remains to be explained. As mentioned before, it seems possible that it reflects a carry-over effect from the sequence of conditions which invariably started with the tone. It may also reflect bilateral processing of verbal material which has been indicated by various imaging approaches [19]. The combination of verbal and nonverbal conditions within one experimental session may have blurred rather than elucidated the co-activation of material-specific networks.

Still, greater right- over left-hemispheric generator activity asymmetry was found in the nonverbal conditions and less asymmetry found in the verbal conditions. Moreover, intra-hemispheric coherence patterns showed distinct, hemisphere-specific patterns for verbal (more pronounced left-hemispheric) and nonverbal (more pronounced right-hemispheric coherence) processing. When lateralized coherence patterns were examined by a laterality index, the clearest left-hemispheric coherence focus emerged for the comprehensible sentence and the clearest right-hemispheric coherence focus emerged for the tone. While we had expected a melody induced dominant right-hemispheric activation, a more bilateral activation was found for the melody-like tone sequence. For the scale, there was a shift towards left-hemispheric asymmetry of coherence. An explanation for this finding might be that the 'melody' was constructed to include the tones of the scale which may have resulted in a melody-like tone sequence even though it did not resemble common melodies or songs. This processing of an unfamiliar 'melody' might have activated temporal (left) and spectral (right) processing, as suggested by [28,29], resulting in a more bilateral activation. While a simple tone contains only spectral information, a melody also contains temporal information.

## Conclusions

In sum, the present study demonstrates that the analysis of the synchronization of evoked magnetic steady-state fields in the source space can map neuronal networks (co-)activated during speech comprehension. Our techniques add spatial information to evidence on left-hemispheric areas involved in language processing, and support co-activation or synchronization within complex neuronal networks as a cortical substrate of integration in perception – like speech comprehension.

## Methods

### Subjects

Data of twelve German native speaking subjects (7 female, mean age 25.3 ± 6.3 years) were included in the analysis. (From the 14 subjects, who had participated in the study, data from one subject had to be discarded because of frequent movement artifacts and from another one, who recognized the play-of-words, see below.) It was ascertained by interview that the subjects did not suffer from any language, audiological or neurological dysfunction. Right-handedness was assessed by a modified version of the Edinburgh handedness questionnaire [32] to be 97.1 ± 4.3. Moreover, all subjects reported having first-degree right-handed relatives. None of the subjects reported to be a professional musician and none reported to be particularly involved in hearing or practicing music. Prior to the experimental session, subjects were informed about the procedure and given informed consent forms. After the experiment, each subject received a financial bonus of 15€.

### Material and design

All stimuli were amplitude modulated at 41.5 Hz (sinusoidal amplitude) with a modulation depth of 90%. Verbal stimuli consisted of words composing a sentence. Nonverbal stimuli consisted of tones forming a scale or a tune or a simple tone. A German play-on-words served as the template for the two verbal conditions. In the first case a sentence is spoken without spacing between words and without accents which creates an incomprehensible word string. The German sentence 'Mähn Äbte Heu? Heu mähn Äbte nie! Äbte mähn Gras' means in English 'Do abbots cut hay? Abbots never cut hay, abbots mow lawns'. If pronounced as a string 'MähnÄbteHeuHeumähnÄbtenieÄbtemähnGras' this utterance, due to a lack of non-phonetic context [33], sounds like speech although meaning cannot be inferred. When the sentence is properly pronounced in the second case, the meaning becomes clear and can be used to parse the information at subsequent trials, allowing a listener to comprehend the sound string as a sentence. For the present study, the incomprehensible string-of-word-version was generated synthetically (software: MBROLA) with a female voice and a fundamental frequency of 200 Hz. None of the 12 subjects included in the data analyses knew the play-of-words and were unable to recognize the meaning of the sentence before it was properly articulated and explained.

The three nonverbal conditions comprised of a 600 Hz sinusoidal tone, a descending major scale (C6 B5 A5 G5 F5 E5 D5 C5, 1034 – 517 Hz), and an arrangement of the same tones (C5 E5 G5 C6 A5 F5 D5 B5). All stimuli of all conditions were adjusted to the length of the sentence and lasted for 4419 ms (sample-rate of 16 kHz/16 bit, mono), and each of the five conditions comprised 15 repetitions that were separated by inter-stimulus intervals of 4419 ms. This long inter-stimulus interval allowed the same signal-to-noise ratio for the baseline and the stimulus conditions which should prevent habituation effects on the SSF. Stimuli were adjusted to have the same average loudness by normalizing to root-mean square (RMS) and were presented at 50 dB above the individually assessed hearing threshold balanced for both ears. In each subject, the hearing threshold was assessed by presenting short 600 Hz beeps with ascending and descending intensity. For each subject and ear the mean hearing threshold was determined from the ascending and descending sequence.

### Task and procedure

During the experiment, which lasted about 45 minutes, the subject was seated in a supine position. Subjects were asked to listen carefully to the stimuli, while fixating a point at the ceiling of the chamber in order to avoid head and eye movements. They were further informed that they would be asked questions about the stimuli during the experimental session, and that they should reply by saying 'yes' or 'no'.

All stimuli were presented in blocks with 15 repetitions. Conditions were separated by breaks of about 5 min each. For every subject the experimental session started with the 600-Hz sinus tone (15 repetitions, condition 1), followed by the word string (condition 2). After 5 repetitions, the subject was asked whether he/she understood what he/she was hearing and could reproduce the meaning of the speech. (None of the subjects could.) Subsequently, the stimulus presentation was continued, and the subject was asked again after the 10th and the 15^th ^presentation, whether he/she understood the meaning of the speech (None of them could).

Then, the experimenter entered the room and pronounced the sentence properly and slowly, so that its meaning became clear. Each subject was asked to reproduce the sentence, in order to ascertain that it was properly understood. After the experimenter had left the subject chamber, the experiment continued with condition 3, which comprised the identical physical stimulation as condition 2 differing only in that the subject now listened to the string of words knowing its meaning, Again, the subjects were asked after 5 repetitions, if they could reproduce the meaning of the sentence, which now they all could. Given that once the sentence's meaning is obvious, one can easily grasp the sentence, the sequence of condition 2 and 3 could not be reversed and thus, the sequence of presentation could not be randomized across subjects.

Condition 4 (scale) and 5 (melody-like tone sequence) were arranged in a similar way, in that the subject was asked after 5 repetitions each, whether or not s/he perceived the sequence of tones as a melody. Eleven of the twelve subjects indicated that the tone sequence sounded like a melody and one was not sure about it. None of them perceived the scale or the simple tone as melodic.

### Data acquisition and analysis

The magnetoencephalogram (MEG) was recorded with a 148-channel whole head system (MAGNES^® ^2500WH, 4D Neuroimaging, San Diego, USA) installed in a magnetically shielded room (Vaccumschmelze, Hanau, Germany). Data were recorded continuously with a sampling-rate of 1017.25 Hz and a 0.1–100 Hz band-pass filter. The electrooculogram (EOG) and the electrocardiogram (EKG) were recorded and stored together with the MEG-data for offline artifact control. Silver-silverchloride electrodes were placed on the outer canthi for the monitoring of horizontal eye movements, and above and below the right eye for vertical eye movements. EKG electrodes were placed on the right collarbone and below left costal arch.

Prior to data analysis, the trials for each condition were submitted to a noise-reduction procedure that subtracted the external noise recorded by MEG reference channels. These noise-corrected data were then bandpass filtered (28–60 Hz, 48 db/Oct, zerophase) and averaged across epochs separately for each condition (epoch-length: 8838 ms, 4419 ms pre-stimulus baseline). Epochs were visually inspected for EOG and EKG artifacts and epochs with magnetic fields greater than 5 pT were rejected. A minimum 13 (of the total 15) epochs per subject were available for further analyses.

The steady state field (SSF) in response to the 41.5-Hz amplitude modulated stimuli was extracted using a moving average procedure. A window of 5 cycles (120.5 ms) of the 41.5 Hz Steady-State signal was shifted 179 times cycle-by-cycle (24.5 ms) across averaged epochs (separately for the 4419-ms baseline and the 4419-ms stimulus duration, the moving average procedure starting 144.5 ms post stimulus). The resulting moving-average epoch was detrended. Figure 1 illustrates that a SSF was successfully induced by the stimulation.

The generators of the SSF were determined in the source space for each epoch using the minimum norm estimate, MNE [34-37] using an algorithm implemented in MATLAB-based in-house software developed by Hauk [35,36].

The MNE is an inverse method reconstructing the primary current that underlies an extracranially recorded time-locked magnetic field. The procedure is based on the assumption that the data vector d, which contains the recorded magnetic activity at given sensor sites, can be described as the product of the leadfield matrix L, which specifies the sensor's sensitivity to the sources, the source current vector j [34] and a noise component ε. Since L and d are known, and ε is treated as if estimated with an accuracy of ~.05, the MNE for j is the mathematically unique solution of the equation which minimizes the squared current density (j^2 ^= min). This solution is obtained by multiplying the pseudo-inverse of the leadfield matrix L with the data. Given the high number of sensors and the presence of noise, spatial regularization is performed with the factor λ. This algorithm allows sources to be omitted, if they do not contribute to the measured magnetic field. A priori information about the number or locations of cortical sources is not required. Following Hauk et al. [35,36], who evaluated the dependence of the accuracy of inverse solutions on the depth of the source for concentric shells, solutions for a shell at 60% radius were determined as a compromise between blurring and depth sensitivity (ca. average radius of cortex, 77 equidistant dipole locations, covering the lateral surface of the brain, were chosen). That is, voltage data were projected to a source space consisting of 350 evenly distributed dipoles with three orthogonal orientations at each dipole location. For every location two tangentially orientated dipole-components were included in further analysis. The mean MNE amplitude, corresponding to the dipole strength in nAm/cm^2^, was determined as mean vector length of both tangentially orientated dipole-components across 5 cycles.

Co-activation of generators was evaluated by all possible pair-wise combinations of the MNE at all dipole locations, according to the algorithm (Matlab, Mathworks):


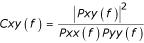


Spectral coherence is a function of frequency with values between 0 and 1 that indicate how well the input x (in the present study MNE at dipole location x) corresponds to the output y (MNE at dipole location y) as a function of frequency (in the present study 41.5 Hz). This algorithm estimates the coherence of two vectors x and y by computing the ratio of the squared cross power spectra (Pxy), divided by the product of the power spectra for each vector (Pxx.*Pyy), where Pxy(f) is the cross power spectrum estimate, Pxx(f) is the power spectrum of the time series at location x, Pyy(f) is the power spectrum estimate of time series at location y and f is the frequency index.

The vectors of 4495 points in length were subdivided into 8 overlapping segments of 613 points (603 ms), each of which was submitted to Hanning windowing. For each vector, the power spectra were obtained as the product of the Discrete Fourier Transforms and its complex conjugate, scaled by the number of points used for x and y, and averaged across segments [38]. For cross spectra the products of discrete Fourier Transforms for vectors x and y were averaged across segments. This algorithm was applied to four pairs of dipole orientations (dpo), normalized (Fisher Z-transformation) and averaged to result in one coherence measure for every pair of locations (dp_x _-dp_y_). As a measure of co-activation or coherence, the first order coherence between a region of interest (ROI) covering 7 locations over Heschl's gyrus and all other 77 locations was determined.

Effects of the five conditions on the distribution of MNE amplitudes and on the coherence measure were evaluated by means of repeated measurement analyses of variance (ANOVA) with the factors CONDITION, HEMISPHERE (comparing all left and all right dipole-locations, excluding midline locations), and GRADIENT (comparing left- and right-anterior versus left- and right- posterior dipole-locations, excluding midline locations). For inspection of the hemispheric asymmetry of MNE, the ANOVA was performed on the Laterality Index (LI: left- minus right-hemispheric MNE divided by their sum, resulting in an index without units). For the evaluation of intra-hemispheric coherence, the first order coherence between the respective left- or right-hemispheric ROI and the other 34 locations of the respective hemisphere entered the ANOVA (comparing conditions), for the evaluation of inter-hemispheric coherence, coherence between the left-hemispheric ROI and all other 34 locations of the right hemisphere and between the right-hemispheric ROI and all other 34 locations of the left hemisphere was submitted to the ANOVA comparing conditions. A separate ANOVA of the two verbal conditions with the factors CONDITION, HEMISPHERE and GRADIENT probed the hypothesis of a change in coherence-topography induced by sentence comprehension. Where appropriate, significance levels are reported with Greenhouse-Geisser correction adjusted degrees of freedom. Interactions were verified by planned posthoc comparisons (two-tailed paired t-tests), and displayed in t-maps without additional alpha correction.

## List of abbreviations

ANOVA: Analysis of variance

EEG: Electroencephalogram

MEG: Magnetoencephalogram

MNE: Minimum Norm Estimate

RMS: Root-mean square

SSF: Steady-State- (magnetic) Field

SSR: Steady-State-Response

## Authors' contributions

MH developed the experimental design, carried out the experimental study and developed and accomplished the data analyses, BR supervised the study and composed the paper, AK advised and assisted the SS-design and SSF analysis, CW supervised the MEG measurements and advised the coherence analyses, TE provided the experimental idea, advised the experimental design, the MEG methods and analyses.
